# Decoding an olfactory mechanism of kin recognition and inbreeding avoidance in a primate

**DOI:** 10.1186/1471-2148-9-281

**Published:** 2009-12-03

**Authors:** Marylène Boulet, Marie JE Charpentier, Christine M Drea

**Affiliations:** 1 Department of Evolutionary Anthropology, 108 BioSci BLDG, Box 90383, Duke University, Durham, North Carolina, 27708, USA; 2 CEFE-CNRS, 1919 Route de Mende, 34293 Montpellier Cedex 5, France; 3 Department of Biology, 108 BioSci BLDG, Box 90383, Duke University, Durham, North Carolina, 27708, USA; 4 Department of Biology, Bishop's University, 2600 College Street, Sherbrooke, Québec, J1M 1Z7, Canada

## Abstract

**Background:**

Like other vertebrates, primates recognize their relatives, primarily to minimize inbreeding, but also to facilitate nepotism. Although associative, social learning is typically credited for discrimination of familiar kin, discrimination of unfamiliar kin remains unexplained. As sex-biased dispersal in long-lived species cannot consistently prevent encounters between unfamiliar kin, inbreeding remains a threat and mechanisms to avoid it beg explanation. Using a molecular approach that combined analyses of biochemical and microsatellite markers in 17 female and 19 male ring-tailed lemurs (*Lemur catta*), we describe odor-gene covariance to establish the feasibility of olfactory-mediated kin recognition.

**Results:**

Despite derivation from different genital glands, labial and scrotal secretions shared about 170 of their respective 338 and 203 semiochemicals. In addition, these semiochemicals encoded information about genetic relatedness within and between the sexes. Although the sexes showed opposite seasonal patterns in signal complexity, the odor profiles of related individuals (whether same-sex or mixed-sex dyads) converged most strongly in the competitive breeding season. Thus, a strong, mutual olfactory signal of genetic relatedness appeared specifically when such information would be crucial for preventing inbreeding. That weaker signals of genetic relatedness might exist year round could provide a mechanism to explain nepotism between unfamiliar kin.

**Conclusion:**

We suggest that signal convergence between the sexes may reflect strong selective pressures on kin recognition, whereas signal convergence within the sexes may arise as its by-product or function independently to prevent competition between unfamiliar relatives. The link between an individual's genome and its olfactory signals could be mediated by biosynthetic pathways producing polymorphic semiochemicals or by carrier proteins modifying the individual bouquet of olfactory cues. In conclusion, we unveil a possible olfactory mechanism of kin recognition that has specific relevance to understanding inbreeding avoidance and nepotistic behavior observed in free-ranging primates, and broader relevance to understanding the mechanisms of vertebrate olfactory communication.

## Background

Most vertebrates recognize their close relatives (kin recognition), either to avoid mating with them or to identify the most appropriate recipients of nepotistic behavior (kin discrimination) [1-3]. Although the benefits of kin recognition may be clear, the mechanisms by which it operates are often less evident. In primates, for instance, researchers typically credit associative, social learning in the discrimination of familiar kin [2,4], but the discrimination of unfamiliar kin (e.g. [5,6]) defies explanation via associative learning [7]. As sex-biased dispersal cannot consistently prevent kin encounters, particularly in long-lived species, inbreeding between unfamiliar kin remains a real threat and carries potentially disastrous fitness consequences [8-10]; consequently, mechanisms to avoid it beg explanation. Indeed, a deeper understanding of communicatory signals demands integration of 'why' questions about ultimate function with 'how' questions about proximate mechanisms [11]. As olfactory-mediated kin discrimination is gaining appreciation in other taxonomic groups [3,12,13], we propose that primates might also use odor cues to assess kinship or genetic relatedness, particularly to identify unfamiliar kin. As a first step to addressing this question, we merge biochemical and genetic analyses to test if olfactory signals offer a reliable means of kin recognition in a strepsirrhine primate, the ring-tailed lemur (*Lemur catta*).

Research on kin discrimination is typically focused on documenting its occurrence. For instance, under natural conditions, researchers have coupled field observations with genetic analyses to show non-random spatial associations or preferential treatment between unfamiliar kin [7,14,15] and avoidance of unfamiliar relatives as mates [16-19]. In the laboratory, researchers have relied on behavioral bioassays or cross-fostering experiments to assess an individual's ability to differentiate unfamiliar kin from non-kin [3]. Here, we focus instead on deciphering a putative olfactory mechanism of kin recognition by showing odor-gene covariance, which occurs when the similarity between the olfactory signals of two individuals reflects their genetic similarity at specific or multiple loci [20]. In inbred mouse lines, for example, at least two gene families (e.g., the major histocompatibility complex or MHC and mouse urinary proteins or MUPs) influence the olfactory profiles of an individual's urine [21-23] and underlie conspecific recognition [24]. Only a handful of studies have begun to examine if similar results might obtain in non-model vertebrates that have different signaling systems or if additional genes may be involved in creating individual odor profiles. Indeed, the interaural secretions of bats relate to maternal lineages [25], the anal secretions of beavers encode pedigree relationships [26], and the scrotal secretions of male ring-tailed lemurs reflect individual genetic diversity (i.e., neutral, whole-genome heterozygosity) and genetic relatedness among males [27]. The latter results have significant implications for olfactory-guided female mate choice and male-male competition, suggesting that odor-gene covariance in this species merits further investigation. Here, we complement our prior findings by examining if the olfactory cues common to female and male genital secretions relate to genome-wide relatedness within and, more importantly, between the sexes. If so, olfactory cues could provide a reliable mechanism of kin recognition to guide nepotistic behavior and inbreeding avoidance.

Ring-tailed lemurs live in multi-male multi-female groups, characterized by a promiscuous breeding system, as well as by female philopatry and male-biased dispersal [28,29]. As a long-lived species that also experiences female eviction [30] and repeated male migration [29], they face the risk of consanguineous mating with unfamiliar kin. When it occurs, inbreeding may have dire consequences, including depressed immune function and reduced life expectancy [31]. Olfactory communication is critical to lemur social interaction, as evidenced by their unique set of specialized scent glands or glandular fields, their elaborate scent-marking repertoire, and the intensity of response these scent signals elicit from conspecifics [28,32-34]. Here, we focus on the genital secretions derived from the labial glandular fields and the scrotal glands because, beyond encoding identity, these secretions are the most comparable between the sexes [34,35].

We sampled 17 sexually mature females year round, during the extended nonbreeding season and the relatively limited breeding season, following published protocols [35]. Our comparable male data (on n = 19 adults) derived from a prior study [27]. We used a sequential approach to determine if genital secretions encode information about relatedness within and between the sexes. For the first analysis involving all female-female (FF) dyads, we related differences in the semiochemical secretions between dyads of females to their pairwise genetic distance. This analysis is particularly relevant to examining olfactory mechanisms guiding nepotistic or competitive behavior between members of the same sex. For the second analysis involving all mixed-sex (MF) dyads, we related differences between the semiochemical secretions and pairwise distances in MM, FF, and MF dyads using a subset of semiochemicals shared by the sexes. For the last analyses of odor-gene covariance, we focused exclusively on MF dyads. These latter analyses are of primary relevance to examining olfactory mechanisms of inbreeding avoidance.

## Results

### Sex differences and similarities in signal composition

**Figure 1:**
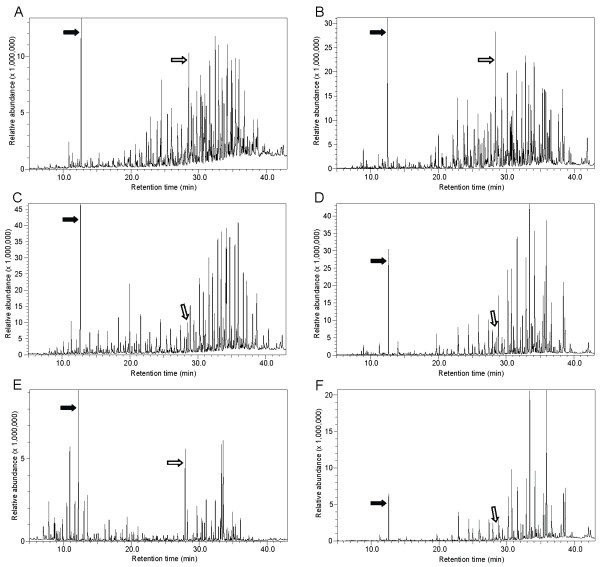
**Odor profiles of the genital secretions in mixed-sex pairs of related and unrelated lemurs**. Chromatograms of labial (first column) and scrotal secretions (second column) from three female-male pairs of lemurs during the breeding season. Top row: maternal half-siblings, representing a sister (A) and her brother (B). For this pair, D_ID _= 0.28 and D_C _= 0.47. Middle row: mother (C) and son (D). For this pair, D_ID _= 0.18 and D_C _= 0.54. Bottom row: unrelated female (E) and male (F) born in different colonies. For this pair, D_ID _= 0.55 and D_C _= 1.16. The filled arrow points to the internal standard, hexachlorobenzene, added to the sample before the GCMS run and the open arrow points to the endogenously produced standard, squalene.

Females expressed a greater number of semiochemicals in their genital secretions than did males (total compounds overall: females = 338, males = 203; mean ± SD compounds in the breeding season: females = 217.8 ± 20.0, males = 135.0 ± 13.5; t-test, t_34 _= 14.7, *P *< 0.001; mean ± SD compounds in the nonbreeding season: females = 200.1 ± 19.3, males = 154.4 ± 10.5; t-test for unequal variance, t_24 _= 8.7, *P *< 0.001). This sex difference was typically accounted for by the presence in females of additional low-molecular weight compounds, such as fatty acid alcohols. Despite this sex difference in overall expression and despite their distinct anatomical derivation [34], labial and scrotal secretions shared a total of 170 compounds, defined as those compounds expressed in at least one female and one male (representing 50.3% and 83.7% of total female and male compounds, respectively; Figure 1). These shared compounds included fatty acid esters (49%), unknown hydrocarbons with identified molecular mass (25%), fatty acids (10%), compounds related to cholesterol (2%), long-chained alcohols (2%), squalene (< 1%), farnesol (< 1%), and 1,6,10-dodecatriene,7,11-dimethyl-3-methylene (< 1%).

### Seasonal sex differences in semiochemical diversity

The sexes also differed in their seasonal patterns of semiochemical diversity, as reflected by three indices, including Richness (which refers to the number of observed semiochemicals) and the Simpson and Shannon indices (which both correct for semiochemical abundance, see Methods and [36]). In males, genital scent signals showed a consistent decline in semiochemical diversity, across all indices, from the nonbreeding to the breeding season [27]. By contrast, the semiochemical diversity of female scent secretions either increased during the breeding season (Richness index, paired t-test, t_16 _= 2.43, *P *= 0.03) or remained stable across seasons (Shannon index: t_16 _= 1.25, *P *= 0.23; Simpson index: t_16 _= 0.27, *P *= 0.79), potentially because those compounds gained during the breeding season contributed only a small proportion (less than 0.5%) to the total chromatogram area. Thus, during the breeding season, whereas signal complexity was compromised in most males, females maintained or enhanced their signal complexity.

### Within-sex odor-gene covariance

**Figure 2:**
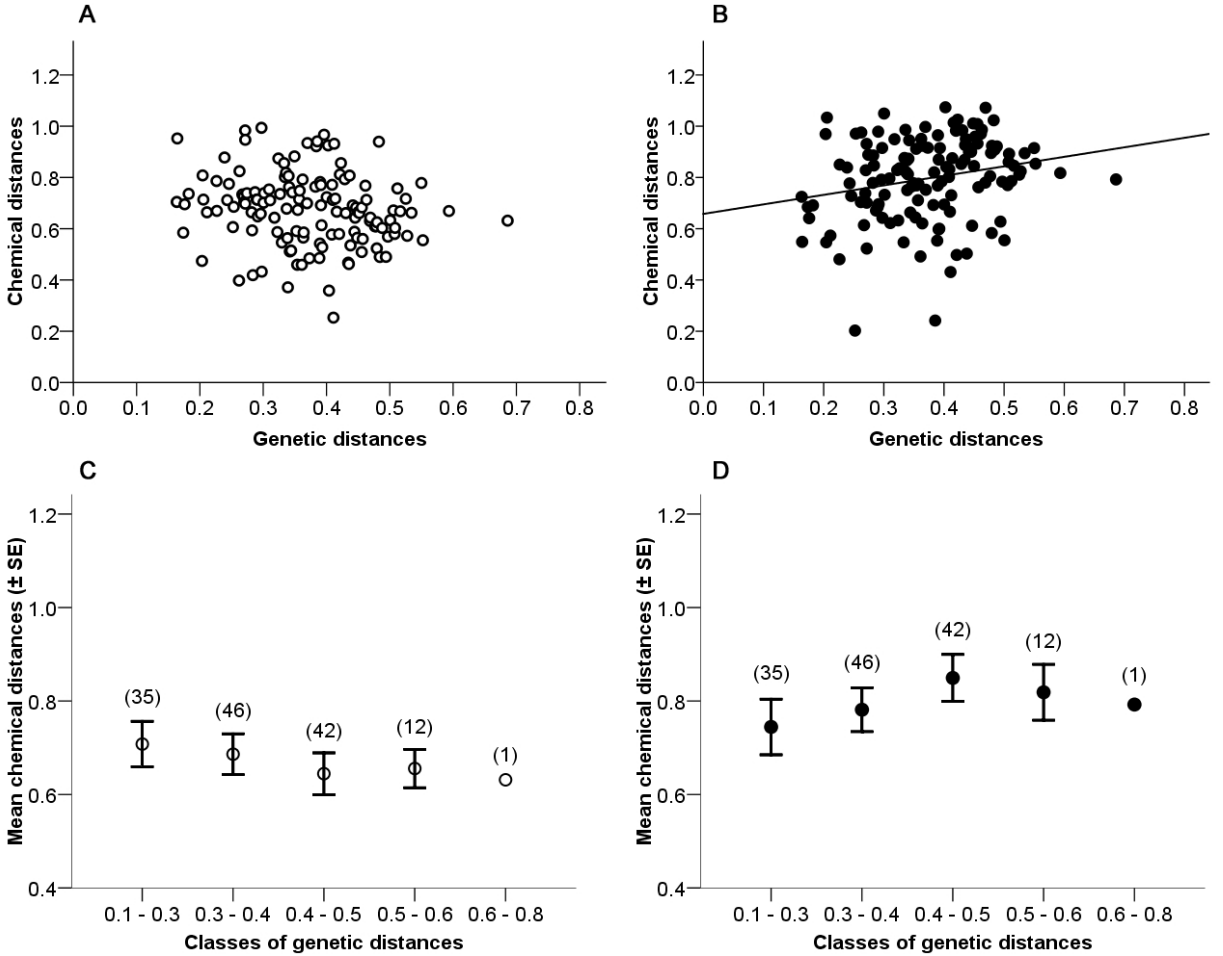
**Odor-gene covariance in female-female dyads**. Relationships between genetic distances (D_ID_) and chemical distances (relative Euclidean, from 338 compounds) in 136 female-female dyads during the nonbreeding (open circles, A and C) and breeding (filled circles, B and D) seasons. The partial Mantel tests were performed on the data illustrated in A and B. For ease of representation and continuity with [27], in C and D we also show these relationships using mean chemical distances per class of genetic distances.

We found that, complementing the pattern in males [27], the olfactory cues present in female genital secretions encoded information about female relatedness in a seasonally dependent fashion. In particular, the chemical distance between members of FF dyads showed no relationship to their genetic distance during the nonbreeding season (partial Mantel test, r = -0.07, *P *= 0.39; Figure 2A and 2C), but correlated significantly with their genetic distance during the breeding season (partial Mantel test, r = 0.22, *P *= 0.02; Figure 2B and 2D).

### Between-sex odor-gene covariance

Representative chromatograms of related and unrelated MF pairs during the breeding season illustrate the greater semiochemical similarity between, for example, brother-sister and mother-son pairs than between two unrelated individuals born to different colonies (Figure 1). To statistically examine the relationship between chemical and genetic distances between mixed-sex pairs, we first ran a partial Mantel test, as above, but for all possible dyads (n = 630 MM + FF + MF dyads). Overall, chemical and genetic distances did not correlate during the nonbreeding season (partial Mantel test, r = 0. 01, *P *= 0.76), but did correlate during the breeding season (partial Mantel test, r = 0.13, *P *= 0.002), consistent with the patterns found independently for same-sex analyses (males: [27]; females: Figure 2).

**Figure 3:**
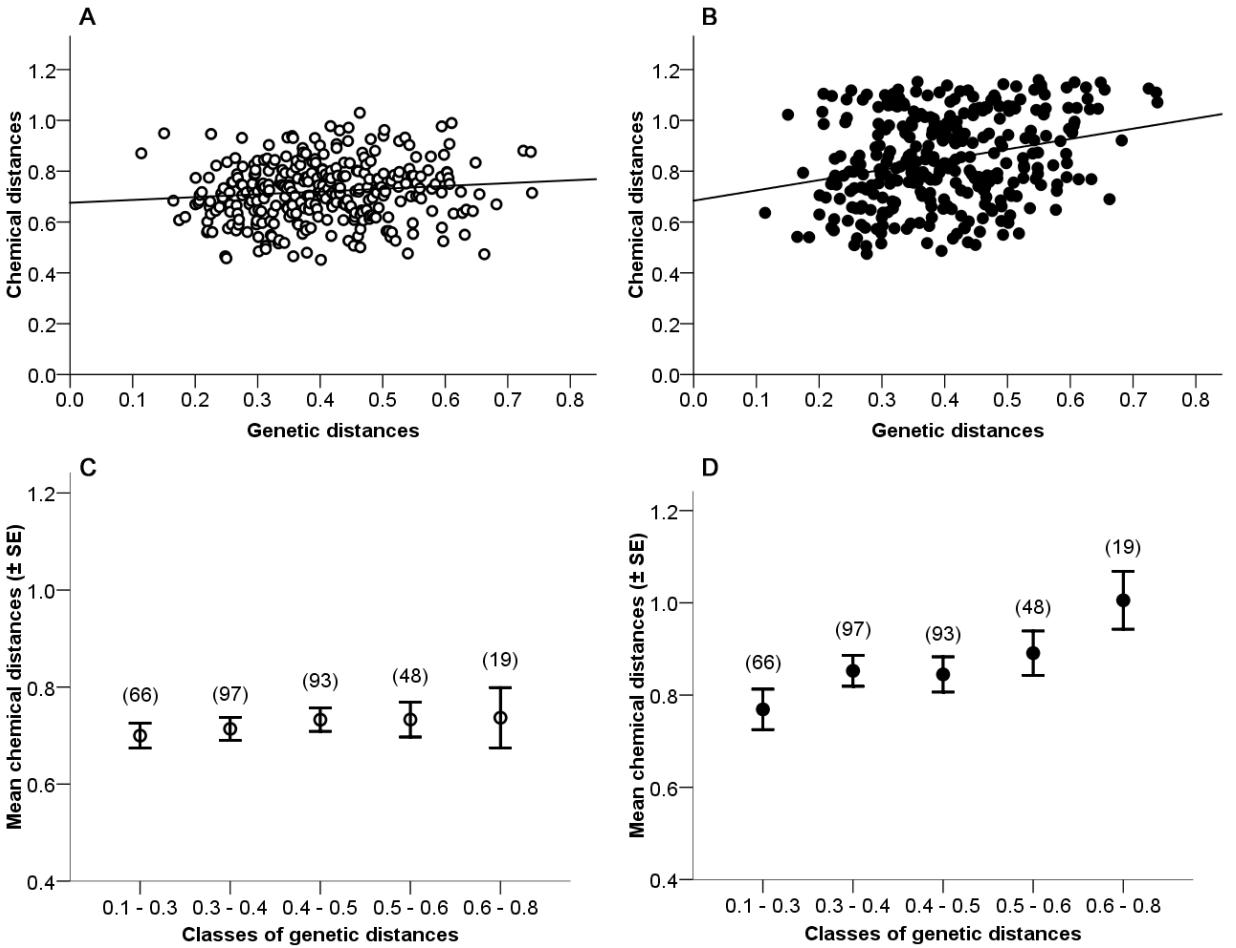
**Odor-gene covariance in male-female dyads**. Relationships between genetic distances (D_ID_) and chemical distances (relative Euclidean, from 170 compounds) in 323 male-female dyads during the nonbreeding (open circles, A and C) and breeding (filled circles, B and D) seasons. The permutation tests on resampling events were performed on the data illustrated in A and B. The permutation tests based on class means were performed on the data illustrated in C and D, which represent mean chemical distances per class of genetic distances, as defined in [27]. The maternal half-siblings (Figures 1 A-B) and the mother-son (Figures 1 C-D) pairs belong to the first category of genetic distances (D_ID _= 0.1 to 0.3), whereas the unrelated pair (Figures 1 E-F) belong to the fourth category of genetic distances (D_ID _= 0.5 to 0.6).

Next, we extracted from the 630 dyads those pairs involving males and females only (n = 323 MF dyads, Figure 3A and 3B). As we could not use the Mantel test for the MF dyads alone (because the corresponding MF matrix would not have been square), we instead used a permutation test to compare the empirical correlation coefficient (Spearman's r) against a distribution of correlation coefficients generated by resampling events [37]. With this analysis, we detected a significant pattern of odor gene-covariance during both the nonbreeding (r = 0.12, *P *= 0.018) and the breeding (r = 0.24, *P *= 0.001) seasons. According to these tests, the odor-gene covariance appeared to be expressed in mixed-sex pairs throughout the year, but was stronger during the breeding season. For a more conservative analysis, we categorized the 323 MF dyads into five classes of genetic distances (Figure 3C and 3D, as for FF dyads in Figure 2C and 2D and as for MM dyads in [27]), which we compared using permutation tests based on class means [38]. During the nonbreeding season, the mean semiochemical distance between members of MF dyads did not vary across classes of genetic distance (all pairwise *P*s > 0.05); olfactory cues in the nonbreeding season did not reflect the genetic relationship between males and females (Figure 3C). During the breeding season, however, the mean semiochemical distances between members of MF dyads were significantly differentiated across nearly all classes (pairwise *P*s < 0.001, except for class 0.3-0.4 vs. class 0.4-0.5, *P *= 1.0), increasing systematically with genetic distance (Figure 3D). According to this analysis, and consistent with within-sex patterns (Figure 2, [27]), the olfactory cues encoding relatedness between the sexes were evident only during the breeding season.

## Discussion

By integrating genetics and biochemistry, we provide the first molecular evidence that the scent secretions expressed by the genital glands of male and female lemurs contain olfactory markers of genetic relatedness within and, more importantly, between the sexes. To date, convergence in olfactory profiles between relatives had been reported only for same-sex dyads [26,27]. Moreover, although semiochemicals common to both sexes have been identified in the secretions derived from the same gland in other species [12,13], we report on signal convergence in the secretions derived from comparable, but anatomically distinct glands or glandular fields [34,39]. Lastly, although the sexes diverged seasonally with respect to their signal complexity, mixed-sex (and same-sex) convergence in the olfactory signals of related individuals appeared most strongly (or only) during the breeding season, suggesting that genital secretions are 'mutual signals' [40] that encode information primarily relevant to inbreeding avoidance, with potentially secondary or independent implications for facilitating nepotism year round. The convergence of olfactory signals within and between the sexes likely underlines strong selective pressures on kin recognition.

Whereas for many females the benefit of inbreeding avoidance may be clear (e.g. to avoid the high energy investment of pregnancy and lactation if the offspring produced would be of low quality), the benefit to males may be less apparent, given their seemingly reduced investment in reproduction. What then might be the selection pressures operating on males to explain olfactory convergence between the sexes? In some species, the olfactory profiles of certain males converge on those of the females to dupe conspecifics in sex communication [41-43]. Such duplicity is unlikely, however, in members of individualized societies. Moreover, it is not supported in ring-tailed lemurs by olfactory evidence, given that sex and individual identity are chemically encoded [35] and detected [33,34] in scent cues throughout the year. An alternate explanation may be found in a broader application of sexual selection theory. Increasing evidence of female-female competition and male mate choice suggests that various species may not conform to a dichotomized pattern of 'expensive eggs' and 'cheap sperm' [44]. Indeed, in socially complex species characterized by slow life histories, males may face significant reproductive costs that increase the benefits derived from being choosy. Thus, female *and *male lemurs may share similar selection pressures to avoid inbreeding.

Odor-gene covariance also raises the possibility that odor cues could serve to prevent outbreeding, again with similar benefits to both sexes. Outbreeding depression, although less well documented than inbreeding depression, can also produce negative consequences on fitness [45,46]. In only a few studies have researchers tested if odorants offer reliable cues that could serve to identify an optimally dissimilar mate and thereby help individuals avoid the fitness consequences of either inbreeding or outbreeding. In humans, individuals tend to pair with mates that are not too dissimilar at functional loci [47], but the sensory mechanism that may drive the avoidance of dissimilar mates remains unclear. In more extreme cases, odor-gene covariance could facilitate species or subspecies recognition. In callitrichid primates, for instance, the scent gland secretions from two subspecies (*Saguinus fuscollis fuscollis *and *S. f. illigeri*) contained distinctive chemical markers [48] and elicited discriminative responses by members of either subspecies [48,49]. Thus, it may be possible that odor-gene covariance mediates outbreeding avoidance within and between species [20]. Given that *Lemur catta*'s nearest sister taxon belongs to a different genus (*Hapalemur*) [50], the threat of hybridization would seem less likely than the threat of inbreeding. Future studies could address the issue of outbreeding by comparing the scent gland semiochemistry of individuals derived from several populations or subspecies and measuring the behavioral responses the various mixtures of semiochemicals may elicit [20].

Although fascinating, it is unclear why the signal of genetic relatedness appears to be largely seasonally dependent. Consistent with the scent secretions of other mammals [51,52], including other primates [48,53,54], the labial and scrotal secretions of ring-tailed lemurs are extremely complex [35] and encode multiple messages [34]. Our permutation analyses suggest that the message of genetic relatedness between males and females may exist during the nonbreeding season also, but as a weaker signal. Perhaps, during the extended nonbreeding season, the message of genetic relatedness may become masked by the expression of compounds encoding other messages. Nonetheless, it could facilitate nepotism year round: Behavioral bioassays show that certain discriminatory responses of lemurs to the scent of conspecifics are limited to the breeding season and may be related to the selection of an appropriate mate, whereas others occur year round and may be related to nepotism (Charpentier MJE, Crawford JC, Boulet M, Drea CM: Lemurs Detect the Genetic Relatedness and Quality of Conspecifics via Olfactory Cues, submitted). Although we have not yet identified any specific semiochemicals responsible for broadcasting genetic relatedness, we have identified seasonal variation in semiochemical expression [27].

Also unclear is the manner by which olfactory signals come to represent an individual's genome. One possible mediating mechanism implicates genetic polymorphism in the enzymes involved in the biosynthesis of semiochemicals. For instance, semiochemical diversity could result from the action of desaturases [55,56] or elongases [57] that modify hydrocarbon-based semiochemicals. While deciphering the biosynthetic pathways of insect pheromones [58], researchers have linked an individual's olfactory profile to the activation of various enzymes [56,58] and have shown that semiochemicals, such as long-chained hydrocarbons, play a role in kin recognition and inbreeding avoidance [57-60]. As most of the semiochemicals found in lemur genital secretions are not present in their monkey chow (Sacha C, Dubay G, Boulet M, and Drea CM; unpublished data), they appear to be endogenously produced and may be derived from common biosynthetic pathways (e.g., squalene is a precursor of cholesterol). Notably, the secretions of lemurs [35] share the diverse hydrocarbon-based semiochemicals found in other mammals [52]. Therefore, the enzymes involved in the synthesis of polymorphic semiochemicals could reflect genome-wide variation and be involved in mammalian chemical communication of genetic relatedness.

Alternatively, or in addition to biosynthetic pathways, an individual's semiochemical profile could be linked to the availability of polymorphic binding proteins that modify the bouquet of semiochemicals emitted by scent glands [61]. Lipocalins represent a large class of extracellular proteins that have the property of binding diverse hydrophobic molecules, such as fatty acids and fatty alcohols [62]. The lipocalin family includes MUPs, a group of binding proteins functioning as semiochemical transporters [63] and notably linked to inbreeding avoidance in mice [23]. These binding properties render lipocalins suitable candidate molecules for kin recognition. Interestingly, two MUP-related genes have been recently detected in the genome of the grey mouse lemur, *Microcebus murinus *[64].

Other candidates include the highly polymorphic MHC genes that are known to affect vertebrate mate choice and kin recognition through their influence on individual odor [21,22,65-68], although the pathway from MHC genes to individual odor profiles may not be completely resolved. In ring-tailed lemurs, specific MHC alleles may correlate with the abundance of some fatty acids present in scent secretions [69]. Data of this sort raise the possibility of an association between MHC genes and the pathways producing semiochemicals [67,70]. The relevant signal of genetic constitution could derive from the semiochemical profile alone or in conjunction with these various binding proteins, and could function in primates and other vertebrates to reveal genetic relatedness. The present findings have clear relevance to strepsirrhine primates and other mammals with functional vomeronasal organs (VNO), including platyrrhines (i.e., New World monkeys [71]). Given the functional overlap between the VNO and main olfactory epithelium [63,72], however, these findings are also likely to extend to catarrhines (i.e., Old World monkeys and apes, including humans) that arguably lack a functional VNO [73,74]. Anthropoid primates produce a diversity of odorous substances (including scent-gland secretions, urine, and axillary sweat) [12,52,54], the functional significance of which remains obscure and underappreciated. Nonetheless, human semiochemicals have been implicated in mother-infant recognition [75], recognition of familiar kin or non-kin [76], and possibly quality/compatibility-based mate choice [68,77,78]. We suggest that an olfactory mechanism could also function in various species to explain the scarcity of mating between unfamiliar kin (e.g. [18]), as well as paternal recognition and protection of offspring (e.g. [79-82]).

## Conclusion

We characterized the secretions expressed by the genital glands of male and female ring-tailed lemurs and showed that these chemical compounds encode genetic relatedness not only within the sexes, but even more strongly so between the sexes. We suspect the link between an individual's genome and its olfactory signals could be mediated in two ways: by biosynthetic pathways producing polymorphic molecules and/or by binding proteins modifying the bouquet of semiochemicals emitted by scent glands. We also found that the relationship between odor profiles and genetic relatedness emerges most strongly during the competitive breeding season, when such information would be crucial for preventing inbreeding and/or directing nepotistic or competitive behavior to appropriate recipients. Although behavioral studies will be necessary to verify the animals' sensitivity to chemically encoded information, we suggest that the seasonal convergence of a mutual olfactory signal between two sex-specific scent glands is likely to reflect strong selective pressure on mate choice. Signal convergence within the sexes may arise as a by-product of between-sex convergence, but could function independently to prevent competition or facilitate altruism between unfamiliar relatives. Our novel analytical approach for identifying odor-gene covariance holds significant promise for deciphering mechanisms of semiochemical signaling in vertebrates and calls specific attention to the important, though often neglected, role of olfactory communication in primates.

## Methods

### Subjects and Housing

Our female subjects were 17 reproductively intact adults (2-23 years old). Females and males were housed at the Duke Lemur Center (DLC) in Durham, North Carolina. Housing conditions have been described elsewhere [27,35] and were in accordance with regulations of the United States Department of Agriculture and with the National Institutes of Health Guide for the Care and Use of Laboratory Animals. All research protocols were approved by the Institutional Animal Care and Use Committee of Duke University (protocol #A245-03-07).

### Collection of odorants and chemical analyses

Information about our collection procedure, odorant extraction, and gas-chromatography mass-spectrometry (GCMS) protocols has been provided elsewhere [27,35]. We collected scent samples over a period of years, spanning November 2003 to November 2007. This sampling period allowed us to maximize our subject pool, which otherwise would have been limited by pregnancy, contraception, immaturity, or death. The maximum time between collection and analysis was three years. We have previously verified sample preservation over freezer storage time [35], as have other researchers [83].

We analyzed chromatograms using the software Solution Workstation (Shimatzu Scientific Instruments). We retained semiochemicals that had consistent retention times and accounted for ≥ 0.05% of the area of the total chromatogram. To align the peaks between chromatograms, we standardized semiochemical retention times (rt) against the retention times of two standards: hexachlorobenzene (rt = 12.6 min) and squalene (rt = 28.5 min), a natural constituent of lemur genital secretions [35]. Semiochemicals were identified by comparing mass spectra against the NIST library, retention time of known compounds, and prior tentative identifications [35].

We compared the semiochemical diversity of secretions between seasons by generating the following indices: richness, Shannon, and Simpson [27]. These indices capture different aspects of the diversity present in semiochemical profiles: Whereas the richness index refers to the total number of semiochemicals per chromatogram, the Shannon and Simpson indices weight semiochemicals according to their abundance [36]. As the mathematical equations of the Shannon and Simpson indices are different, they generate distinct biases: the Shannon index is most strongly influenced by the compounds that are of intermediate abundance, whereas the Simpson index is most sensitive to the compounds that show the greatest abundance. All indices were computed in the software PC-ORD 5.20 [36].

For the analyses of odor-gene covariance in FF dyads, we retained 338 semiochemicals (retention time between 8.02 to 42.56 min), which we expressed as peak area relative to the total area of the chromatogram. For similar analyses in mixed-sex dyads (MF), we first compared the 338 semiochemicals detected in labial samples to the 203 semiochemicals previously detected in scrotal secretions [27] to identify those semiochemicals shared by both sexes (i.e., those present in at least one male and one female). We then used only those 170 shared compounds in our mixed-sex analyses. As a proxy for chemical distance (D_C_), per season, we estimated the relative Euclidean distances between the semiochemical profiles of all possible dyads (n = 171 MM dyads; n = 136 FF dyads; n = 323 MF dyads) using the software PC-ORD 5.20 [36] and following previously published protocols.

### Genetic analyses

We genotyped 81 DLC colony members at 11-14 microsatellite loci. These numbers differ slightly from those (i.e., n = 73 individuals at 9-14 loci) previously reported [27], reflecting the additional genotyping of some individuals at certain loci. We used the Identity index, R_ID_, as an estimate of genetic relatedness [84] and transformed this index to obtain genetic distances, D_ID_, using the following equation: D_ID _= 1- R_ID_. We obtained similar results using the Queller and Goodnight index [85]. From all possible pairwise combinations, we retrieved those pairs involving subjects for which we had semiochemical data (partial Mantel test with 17 females: n = 136 FF dyads; partial Mantel test with 17 females and 19 males: n = 630 MM + FF + MF dyads; permutation tests, n = 323 MF dyads).

### Statistical analyses

We compared chemical diversity between male and female secretions by computing t-tests or t-tests for unequal variances (which adjusts the degrees of freedom). To test for seasonal differences in chemical diversity in female secretions, we used a paired t-test (SPSS 15.0 for Windows, SPSS Inc.). We tested for linear relationships between D_C _and D_ID _by computing partial Mantel tests (Fstat version 2.9.3.2; with 2000 randomizations and backward selection of variables, [86]). As in our previous study [27], we controlled for various potentially confounding factors, including the subject's age, their housing conditions, and the month of sample collection (see Additional file 1). For the partial Mantel test on MM + FF + MF dyads, we included sex as a supplementary co-variable (see Additional file 2). The matrices included 171 MM, 136 FF dyads, and 323 MF dyads.

As we were particularly interested in testing if genital scent secretions may encode information that could be used in the context of inbreeding avoidance, we again correlated chemical distances with genetic distances, focusing specifically on MF dyads. We used two types of permutation tests to address this question. For the first type of permutation test [37], we calculated the empirical correlation coefficients (see Additional file 3) between chemical and genetic distances of MF dyads and compared them against a distribution of correlation coefficients generated by 1000 permutation events (Resampling Stats for Excel, version 4.0). For the second type of permutation test, we categorized D_ID _values into 5 classes of genetic distances (0.1 to 0.3; 0.3 to 0.4; 0.4 to 0.5; 0.5 to 0.6; 0.6 to 0.8). These genetic classes follow those defined for the MM dyads by roughly equilibrating sample sizes across categories [27]. We then compared the mean chemical distances between these classes using randomization tests (PERM software, [38]). Each randomization test included 10 iterations of 1000 permutation events and *P *values were averaged across all iterations. We applied sequential Bonferroni corrections to each pairwise comparison [87].

## Authors' contributions

MB participated in the design of the study, carried out chemical analyses, performed statistical tests, and wrote the first draft of the manuscript. MJEC participated in the design of the study, carried out genetic work, performed statistical analyses and participated in drafting the manuscript. CMD conceived the research program in primate olfactory communication, participated in the design of the study and critically revised draft versions of the manuscript. All authors approved the final version of the manuscript.

## Supplementary Material

Additional file 1 **Socio-demographic and environmental factors (female-female dyads only)**. Supplementary information and Table 1. Partial Mantel tests showing the seasonal relationships between semiochemical distances (relative Euclidean distances derived from 338 compounds) of female labial secretions versus genetic distances (D_ID_) and versus three socio-demographic or environmental factors taken as co-variables (n = 136 FF dyads). Click here for file

Additional file 2 **Socio-demographic or environmental factors (all possible dyads)**. Supplementary information and Table 2. Partial Mantel tests showing the seasonal relationships between semiochemical distances (relative Euclidean distances derived from 170 compounds) of lemur genital secretions versus genetic distances (D_ID_) and versus four socio-demographic or environmental factors taken as co-variables (n = 630 MM + FF + MF dyads). Click here for file

Additional file 3 **Permutation tests based on 1000 resampling events, for 630 MM + FF + MF dyads and 323 MF dyads**. Supplementary Table 3. Permutation tests showing the seasonal relationships between semiochemical distances of genital secretions versus genetic distances (D_ID_) for MM + FF + MF dyads (n = 630) and for MF dyads only (n = 323). Click here for file
