# Supplementary material for: Decoding an olfactory mechanism of kin recognition and inbreeding avoidance in a primate
Source: BMC Evol Biol. 2009 Dec 3;9:281. doi: 10.1186/1471-2148-9-281 (PMC2799416; doi:10.1186/1471-2148-9-281)
Supplement: Additional file 1 — Socio-demographic and environmental factors (female-female dyads only). Supplementary information and Table 1. Partial Mantel tests showing the seasonal relationships between semiochemical distances (relative Euclidean distances derived from 338 compounds) of female labial secretions versus genetic distances (DID) and versus three socio-demographic or environmental factors taken as co-variables (n = 136 FF dyads). [file 1471-2148-9-281-S1.DOC]

**Additional file 1**

Supplementary Table 1. Partial Mantel tests showing the seasonal relationships between semiochemical distances (relative Euclidean distances derived from 338 compounds) of female labial secretions versus genetic distances (D_ID_) and versus three socio-demographic or environmental factors taken as co-variables (n = 136 FF dyads).

| Variable | Nonbreeding | | Breeding | |
| --- | --- | --- | --- | --- |
|  | r | *P* | r | *P* |
| D_ID_ | -0.074 | 0.39 | **0.215** | **0.02** |
| Age | -0.031 | 0.91 | -0.049 | 0.96 |
| Housing | -0.015 | 0.87 | -0.005 | 0.96 |
| Month of collection | **0.616** | **< 0.001** | 0.164 | 0.06 |

For age, we calculated the difference (in years) between pairs of subjects. As the lemurs were not always housed in the same social condition (i.e., they could be housed singly or socially, including in all-male groups, all-female groups, one-male groups, or multi-male multi-female groups), we coded these differences between pairs of subjects as follows: 0 for identical housing condition and 1 for different housing condition. For month of collection, we scored the number of months between samples, irrespective of the year (e.g. samples obtained in June 2005 and July 2006 would have a month distance of 1). We included month of collection, but not year of collection for the following reasons. Ring-tailed lemur semiochemistry changes over the annual reproductive cycle, while animals maintain consistent individual ‘signatures’ across years [1]. Thus, relative to season-to-season variation, year-to-year variation is minimal. Moreover, in preliminary analyses, the year of collection was often confounded with other variables, including month of collection and sex. Thus, under the present circumstances, significant effects of year would likely owe to seasonal or individual effects.

The only factor that was related to semiochemical distances during the nonbreeding season was the month of sample collection. The effect was driven by those dyads involving a single female whose samples were collected in a different month from those of the other females. Therefore, there was more annual spread represented for the nonbreeding season than for the breeding season. As the nonbreeding season in the Northern Hemisphere lasts approximately 8 months (early March to end of October) and the breeding season occurs as consecutive, relatively synchronized cycles during a 4-month period, the collection effect we detected may reflect significant seasonal variations in female semiochemistry within the relatively long nonbreeding season (which here encompasses the prebreeding season), as was previously documented [1]. In the breeding season, no factors other than genetic distance correlated with semiochemical distances.

**References**

1. Scordato ES, Dubay G, Drea CM: **Chemical composition of scent marks in the ringtailed lemur (*Lemur catta*): glandular differences, seasonal variation, and individual signatures.** *Chem Senses* 2007, **32:**493-504.
