# Supplementary material for: Decoding an olfactory mechanism of kin recognition and inbreeding avoidance in a primate
Source: BMC Evol Biol. 2009 Dec 3;9:281. doi: 10.1186/1471-2148-9-281 (PMC2799416; doi:10.1186/1471-2148-9-281)
Supplement: Additional file 2 — Socio-demographic or environmental factors (all possible dyads). Supplementary information and Table 2. Partial Mantel tests showing the seasonal relationships between semiochemical distances (relative Euclidean distances derived from 170 compounds) of lemur genital secretions versus genetic distances (DID) and versus four socio-demographic or environmental factors taken as co-variables (n = 630 MM + FF + MF dyads). [file 1471-2148-9-281-S2.DOC]

**Additional file 2**

Supplementary Table 2. Partial Mantel tests showing the seasonal relationships between semiochemical distances (relative Euclidean distances derived from 170 compounds) of lemur genital secretions versus genetic distances (D_ID_) and versus four socio-demographic or environmental factors taken as co-variables (n = 630 MM + FF + MF dyads).

| Variable | Nonbreeding | | Breeding | |
| --- | --- | --- | --- | --- |
|  | r | *P* | r | *P* |
| D_ID_ | 0.013 | 0.76 | **0.130** | **0.002** |
| Age | 0.033 | 0.51 | - 0.016 | 0.51 |
| Housing | - 0.017 | 0.66 | **0.109** | **0.01** |
| Month of collection | **0.086** | **0.04** | **0.085** | **0.04** |
| Sex | **0.471** | **< 0.001** | **0.531** | **< 0.001** |

The subject’s age, their housing conditions, and the month of sample collection were coded as explained in Additional file 1. We coded sex differences between pairs of subjects as follows: 0 referred to same-sex dyads and 1 referred to mixed-sex dyads.

Three socio-demographic or environmental factors correlated with semiochemical distances. First, as anticipated, same-sex secretions were more alike than were opposite-sex secretions, regardless of season [1]. Second, during the breeding season, individuals housed in similar conditions (e.g., alone or in groups) produced semiochemical profiles that were more alike than were those of individuals housed in different conditions, suggesting that the social environment might influence individual odors in lemurs, as it does in other species [2, 3]. Here, social environment acted synergistically with genetic background, enhancing the odor differentiation between individuals housed in different conditions. Lastly, samples of scent secretion collected in the same month were more alike than were those collected several months apart, consistent with previously reported seasonal differences [1].

**References**

1. Scordato ES, Dubay G, Drea CM: **Chemical composition of scent marks in the ringtailed lemur (*Lemur catta*): glandular differences, seasonal variation, and individual signatures.** *Chem Senses* 2007, **32:**493-504.

2. Pohorecky LA, Blakley GG, Ma EW, Soini HA, Bruce KE, Novotny M: **Social housing influences the composition of volatile compounds in the preputial glands of male rats.** *Horm Behav* 2008, **53:**536-545.

3. Havlicek J, Lenochova P: **Environmental effects on human body odour**. In *Chemical signals in Vertebrates 11.* Edited by: Hurst JL, Beynon RJ, Roberts SC, Wyatt TD. New York: Springer; 2008:199-210.
