# Supplementary material for: Decoding an olfactory mechanism of kin recognition and inbreeding avoidance in a primate
Source: BMC Evol Biol. 2009 Dec 3;9:281. doi: 10.1186/1471-2148-9-281 (PMC2799416; doi:10.1186/1471-2148-9-281)
Supplement: Additional file 3 — Permutation tests based on 1000 resampling events, for 630 MM + FF + MF dyads and 323 MF dyads. Supplementary Table 3. Permutation tests showing the seasonal relationships between semiochemical distances of genital secretions versus genetic distances (DID) for MM + FF + MF dyads (n = 630) and for MF dyads only (n = 323). [file 1471-2148-9-281-S3.DOC]

**Additional file 3**

Supplementary Table 3. Permutation tests showing the seasonal relationships between semiochemical distances of genital secretions versus genetic distances (D_ID_) for MM + FF + MF dyads (n = 630) and for MF dyads only (n = 323).

| Correlation coefficient for D_ID_ | MM + FF + MF dyads | | | | MF dyads | | | |
| --- | --- | --- | --- | --- | --- | --- | --- | --- |
|  | Nonbreeding | | Breeding | | Nonbreeding | | Breeding | |
|  | r | *P* | r | *P* | r | *P* | r | *P* |
| Spearman’s, ranked data | - 0.0001 | 0.485 | **0.105** | **0.002** | **0.116** | **0.018** | **0.236** | **0.001** |
| Pearson’s, untransformed data | 0.0087 | 0.423 | **0.113** | **< 0.001** | **0.111** | **0.022** | **0.264** | **< 0.001** |

We calculated empirical correlation coefficients between chemical and genetic distances using the raw data (i.e., Pearson’s r) and ranked-transformed data (i.e., Spearman’s r). Then, we shuffled the empirical chemical distances and calculated the new (or ‘simulated’) correlation coefficients [1]. This shuffling and scoring procedure was repeated 1000 times to generate a distribution of simulated correlation coefficients (Resampling Stats for Excel, version 4.0). To generate a *P* value, we counted how many times the value of the simulated correlated coefficient exceeded the value of the empirical correlation coefficient. We then divided this number by 1000.

**Reference**

1. Simon J. *Resampling: the new statistics*. 2^nd^ Edition. Ebook available at <http://www.resample.com/content/text/index.shtml>. 1997.
